# Primary testicular diffuse large B-cell lymphoma: A case report focusing on touch imprint cytology and a non-germinal center B-cell-like phenotype

**DOI:** 10.3892/etm.2013.1091

**Published:** 2013-04-29

**Authors:** HYUN-SOO KIM

**Affiliations:** Molecular Biology Laboratory, Aerospace Medical Center, Republic of Korea Air Force, Cheongwon-gun, Chungcheongbuk-do 363-849, Republic of Korea

**Keywords:** diffuse large B-cell lymphoma, testis, touch imprint cytology, non-germinal center B-cell-like phenotype

## Abstract

Primary diffuse large B-cell lymphoma (DLBCL) of the testis is a rare subtype of testicular tumor. While the histomorphology of testicular DLBCL is well described, a paucity of information in the literature exists with regard to the cytological diagnosis of this subtype of tumor. Touch imprint specimens were obtained from a testicular DLBCL occurring in a 64-year-old man. The cytological features of imprints were compared with the results obtained from histological and immunohistochemical examinations. Smears obtained from the touch imprints exhibited a high cellular yield consisting of discretely arranged monomorphic large cells with irregular nuclear membranes, scant cytoplasm and conspicuous nucleoli. Histologically, the tumor consisted of discohesive neoplastic lymphocytes that infiltrated diffusely and produced a wide separation of intact seminiferous tubules. Diffuse, intense immunostaining for CD45, CD20, MUM1 and Ki-67 led to the diagnosis of primary DLBCL of the testis with a non-germinal center B-cell-like phenotype. Careful observation of the touch imprint specimens of testicular DLBCL revealed a high cellularity with a predominant single-cell pattern of monomorphic cells demonstrating irregular nuclear membranes and conspicuous nucleoli. In addition, DLBCL is capable of developing in the testis and forming a predominantly discohesive cell population, suggesting the presence of a lymphoid malignancy. Thus, it may be possible to detect morphological features that are characteristic of DLBCL using imprint cytology. To the best of our knowledge, this is the first study reporting the diagnosis of testicular DLBCL using touch imprint cytology.

## Introduction

Primary testicular lymphoma is a collection of neoplasms that constitutes only 1–9% of testicular tumors (1). Although uncommon in the general population, it is the most common type of malignant testicular tumor in men ≥50 years of age (2). There are various subtypes, including diffuse large B-cell lymphoma (DLBCL), Burkitt’s lymphoma and follicular lymphoma. In the adult testis, primary DLBCL represents the most frequent subtype of lymphoma (80–90%), whereas the majority of testicular lymphomas in children consist of secondary involvement by Burkitt’s lymphoma, DLBCL or lymphoblastic lymphoma (3). The typical clinical sign is a painless testicular mass of variable size that is usually unilateral. Primary testicular lymphoma may be identified during the initial presentation of primary or systemic malignant lymphomas, or during a clinical follow-up of patients with lymphoma (4). Historically, primary testicular lymphoma has been reported to exhibit a poor prognosis with an overall 5-year survival rate of 17–48%, particularly primary testicular DLBCL, whose clinical behavior has been reported to be aggressive and to demonstrate a high propensity to disseminate to the central nervous system (CNS) and skin at presentation and relapse (5,6). The underlying mechanisms responsible for this aggressive behaviour have yet to be elucidated.

In the present study, a patient with primary testicular DLBCL was examined and touch imprint specimens from the patient’s tumor were obtained. While the histomorphology of testicular DLBCL is well described, no information with regard to the cytological diagnosis of this tumor is currently available in the literature. Furthermore, the imprint cytological features of primary testicular DLBCL have yet to be reported; thus, our results are considered to be of interest. The imprint cytological findings were compared with those obtained from histological examination and immunohistochemical staining in order to evaluate the significance of touch imprint cytology in the diagnosis of testicular DLBCL.

## Case report

**Figure 1.**
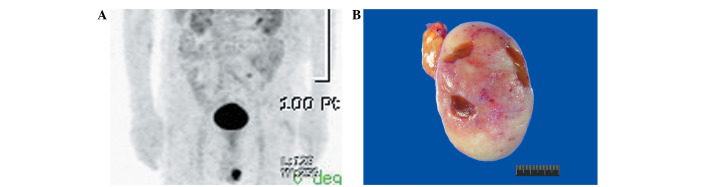
Imaging and gross pathological findings. (A) Positron emission tomography scanning revealed a hypermetabolic lesion in the left scrotum. (B) A left orchiectomy specimen demonstrated a well-circumscribed tumor formation measuring 7.5×5.5×4.8 cm. The testicular parenchyma was completely replaced with a pale yellow-to-pink solid tumor with a fleshy cut surface. Touch imprints were performed in viable tumor areas.

A 64-year-old male presented with a slowly growing, painless enlargement in the left scrotum that was discovered by the patient ∼2 months beforehand. The patient had a history of mild alcohol ingestion, inguinal hernia, benign prostatic hyperplasia and lobectomy due to non-small cell lung carcinoma that had been fully removed 2 years previously. There was no history suggestive of cryptorchidism or any endocrine symptoms. The patient had a heavy feeling in the left scrotum and physical examination revealed a left testicular mass measuring approximately the size of an adult’s fist. The right testicle was normal. The patient had no lymphadenopathy or hepatosplenomegaly. Examination of the oronasopharynx revealed no abnormal results. Laboratory test results, including hematological, urinary and biochemical values, were within normal range. No abnormal results were observed following an abdominal computed tomography scan. Results of a thoracic computed tomography scan were also normal. Positron emission tomography scanning demonstrated a conspicuous hypermetabolic lesion in the left scrotum (Fig. 1A). As a testicular neoplasm or orchitis was clinically suspected, a left orchiectomy was performed.

**Figure 2.**
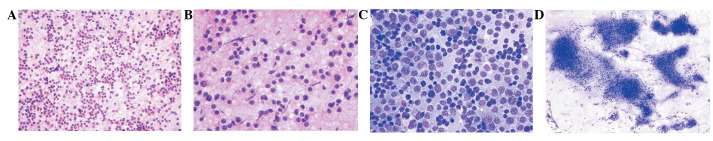
Cytological findings. (A) Smears obtained from touch imprint cytology were highly cellular, consisting of a monomorphic malignant cell population arranged in a predominantly single-cell pattern. (B) The tumor cell nuclei were enlarged and hyperchromatic with conspicuous nucleoli, and had a narrow rim of pale-to-clear cytoplasm with a distinct outer border. A necrotic background suggested the malignant nature of this lesion. (C) A high-power view demonstrated a single-cell arrangement of enlarged nuclei with an irregular nuclear membrane. (D) In some areas, cohesive clusters of tumor cells were detected. (A and B) Hematoxylin and eosin (H&E) staining and (C and D) Wright-Giemsa staining. Original magnification: (A) ×200; (B) ×400; (C) ×600 and (D) ×40.

The resected specimen demonstrated the formation of a well-circumscribed tumor measuring 7.5×5.5×4.8 cm. Grossly, the cut surface of the tumor was solid, fleshy, lobulated and pale yellow-to-pink with hemorrhagic punctuations (Fig. 1B). The tumor exhibited a homogeneous texture and diffusely replaced the testicular parenchyma. The epididymis, spermatic cord and adjacent soft tissues appeared normal. Smears obtained from a touch imprint of the lesion were highly cellular, consisting of discretely arranged monomorphic large cells (Fig. 2A). The individual tumor cells exhibited a high nucleo-cytoplasmic ratio and clear cytoplasm forming a narrow rim around the nucleus and a distinct outer cell border (Fig. 2B). The nuclei were enlarged, clumped and hyperchromatic, with irregular nuclear membranes and conspicuous single to multiple nucleoli (Fig. 2C). Intermingled amongst the large tumor cells were small, round lymphocytes. In some areas, tumor cells arranged in cohesive groups were also detected (Fig. 2D).

**Figure 3.**
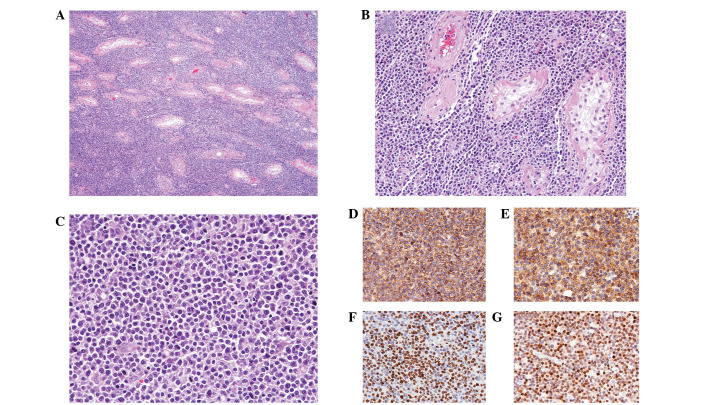
(A–C) Histological and (D–G) immunohistochemical findings. (A) The testicular tumor demonstrated a monomorphic population of neoplastic lymphocytes with a diffuse growth pattern. (B) Tumor cells penetrated diffusely into tissue spaces, producing a wide separation of intact seminiferous tubules. Also observed was tubular hyalinization. (C) Tumor cells demonstrated enlarged nuclei with irregular nuclear membranes and conspicuous nucleoli. Immunostaining revealed that the tumor cells exhibited diffuse, intense reactivity for (D) CD45, (E) CD20, (F) Ki-67 and (G) MUM-1. (A–C) Hematoxylin and eosin (H&E) staining and (D–G) polymer method. Original magnification: (A) ×40; (B) ×200; (C) ×400 and (D–G) ×200.

Histologically, the testicular tumor demonstrated complete replacement by a monomorphic population of neoplastic lymphocytes with a diffuse growth pattern (Fig. 3A). The tumor cells penetrated diffusely into tissue spaces, producing a wide separation of intact seminiferous tubules (Fig. 3B). Spermatogenic arrest, interstitial fibrosis and tubular hyalinization were also observed. Similar to the results observed in the touch imprint specimens, the tumor cells demonstrated enlarged nuclei with irregular nuclear membranes and conspicuous nucleoli (Fig. 3C). In a few areas, there was a destruction of the tubular wall and blood vessel wall with invasion of the lumen. The tumor did not infiltrate the epididymis, spermatic cord, tunica albuginea or tunica vaginalis. Immunohistochemical assays revealed that the tumor cells were markedly positive for CD45 (Fig. 3D) and CD20 (Fig. 3E); however, they were negative for CD3, epithelial membrane antigen and pancytokeratin, indicative of a B-cell lymphoid malignancy. The proliferation index as detected by Ki-67 staining was high (∼90%; Fig. 3F). In addition, since the tumor demonstrated a CD10-negative, MUM1-positive (Fig. 3G) and BCL6-negative immunoprofile, this case was classified as non-germinal center B-cell-like DLBCL (non-GCB-DLBCL) (7). This study was approved by and conducted in accordance with the policies of the Institutional Review Board of Republic of Korea Air Force. Informed consent was obtained from the patient.

## Discussion

Although the greatest accuracy of cytological examination of the testis is observed in azoospermic males whose smears demonstrate normal spermatogenesis, the diagnostic accuracy of cytological examination in testicular neoplasms has also been reported to be extremely high (8,9). Previous studies have described the cytomorphology of numerous types of testicular malignancy, including classic and spermatocytic seminoma, embryonal carcinoma and metastatic lesions (8,10,11); however, no information with regard to the cytological diagnosis of primary testicular DLBCL is available in the literature. To the best of our knowledge, the present study is the first to describe the cytological features of testicular DLBCL. Smears obtained from the touch imprints exhibited a high cellular yield predominantly consisting of discretely arranged monomorphic lymphocytes with irregular nuclear membranes, scant cytoplasm and conspicuous nucleoli. These findings were identical to those of primary nodal DLBCL.

The observation of cohesive cellular aggregates in specific areas of the imprint cytological smear slides was notable. A pattern of cohesive groups of tumor cells is not common in DLBCL, although it may occasionally occur. This atypical feature mimics metastatic carcinoma and may confound diagnosis. In fact, distinguishing DLBCL from metastatic carcinoma on cytological examination is usually a straightforward procedure for an experienced pathologist. Generally, benign or malignant lymphoid cells are characterized by a predominant single-cell pattern on cytology specimens, whereas carcinoma cells typically exhibit cohesive clusters (12). Although lymphoma cells may occasionally artificially demonstrate focal cohesion, particularly in highly cellular specimens, the predominant single-cell pattern in the background usually aids in establishing the correct diagnosis.

Previously, it has been shown that DLBCLs may be divided into three prognostically distinct subtypes by gene expression profiles using a cDNA microarray (7): GCB-DLBCLs, activated B-cell-like DLBCLs and type 3. The immunohistochemical expression of CD10, BCL6 and MUM1 may be used to categorize DLBCLs into GCB and non-GCB types, the latter including activated B-cell-like types and type 3 (13). GCB-DLBCLs are assigned to those that express CD10 and/or are positive for BCL6 but negative for MUM1, and non-GCB-DLBCLs are assigned to those negative for CD10 and positive for MUM1. These subtypes differ in clinical behavior, i.e., GCB-DLBCLs have an improved clinical outcome compared with non-GCB types. Al-Abbadi *et al* (14) demonstrated that primary testicular DLBCL exhibited non-GCB type gene expression almost exclusively. Li *et al* (6) also demonstrated that the majority of primary testicular DLBCLs exhibited non-GCB type characteristics and the overall survival rate of patients with non-GCB-DLBCL was significantly lower compared with patients with GCB-DLBCL. Based on these data, it is reasonable to hypothesize that the main explanation for the poor prognosis of primary testicular DLBCL may be its correlation with the non-GCB phenotype.

Differential diagnosis of primary testicular DLBCL may involve a number of germ cell tumors, including classic seminoma, spermatocytic seminoma and embryonal carcinoma (1). Granulomatous and viral orchitis may also mimic lymphoma histologically. Seminoma cells, unlike the majority of lymphoma cells, have distinct cell membranes, abundant glycogen-rich cytoplasms and rounded but focally flattened central nuclei. The cells of spermatocytic seminoma are polymorphous and belong to three distinct types. Embryonal carcinoma has a characteristic epithelioid appearance that frequently forms glandular, papillary or tubular structures. Lymphomas often possess smaller cells with less cytoplasm and a higher nucleo-cytoplasmic ratio. In addition, they demonstrate diffuse intertubular infiltration with recognizable tubular remnants. This characteristic intertubular growth pattern of lymphoma is initially suggestive of the diagnosis in numerous cases. Furthermore, in contrast to seminoma and embryonal carcinoma, lymphomas lack precursor intratubular germ cell neoplasia. Although lymphoma cells may invade the seminiferous tubules, they do not demonstrate the regular basal alignment within the tubules that is observed in intratubular germ cell neoplasia. Viral and granulomatous orchitis have heterogeneous and benign-appearing inflammatory cellular infiltrates, in contrast to the more homogeneous and malignant-appearing infiltrate of lymphoma.

The treatment for patients with primary testicular DLBCL may be divided into limited disease (stage I/II) and advanced disease (stage III/IV) treatments. For limited disease, a standard treatment has yet to be established (2). Orchiectomy provides histological tissue for diagnosis and also removes a potential sanctuary site, as the blood-testis barrier renders testicular tumors inaccessible to systemic chemotherapy (15). The cyclophosphamide, doxorubicin, vincristine and prednisone (CHOP) regimen has been the mainstay of therapy for several decades. More recently, the addition of the anti-CD20 monoclonal antibody rituximab to the CHOP regimen (R-CHOP) has led to a marked improvement in progression-free and overall survival (16). Routine CNS prophylaxis is recommended in patients with primary testicular lymphoma of any stage due to the high rate of CNS recurrence. Radiation therapy may be used as a prophylactic therapy to prevent relapse in the regional lymph nodes or in the controlateral testis, or to treat lymphomatous lesions, including retroperitoneal lymphadenopathies. For advanced disease, patients should be treated according to the guidelines for the treatment of advanced stage nodal DLBCL. The standard therapeutic option for patients with stage III/IV disease is conventional-dose anthracycline-containing chemotherapy plus rituximab with the addition of prophylactic scrotal radiotherapy and intrathecal chemotherapy. The standard therapeutic option for patients with relapsed disease has yet to be defined in prospective trials. However, the therapeutic strategy should be identical to the strategies used for other relapsed aggressive forms of non-Hodgkin’s lymphoma.

In conclusion, careful observation of the touch imprint specimen of testicular DLBCL reveals a high cellularity with a predominant single-cell pattern of monomorphic cells demonstrating irregular nuclear membranes and conspicuous nucleoli. In addition, taking into consideration that DLBCL is capable of developing in the testis and forming a predominantly discohesive cell population that suggests a lymphoid malignancy, it may be possible to detect morphological features characteristic of DLBCL using imprint cytology. To the best of our knowledge, this is the first study describing the touch imprint cytological diagnosis of testicular DLBCL. It is important to identify primary testicular DLBCL correctly and to distinguish it from other entities due to differences in therapy, management and prognosis.
